# A Multifaceted Intervention to Improve Medication Adherence in Kidney Transplant Recipients: An Exploratory Analysis of the Fidelity of the TAKE IT Trial

**DOI:** 10.2196/27277

**Published:** 2022-05-05

**Authors:** Esther S Yoon, Scott Hur, Laura M Curtis, Aiden H Wynia, Pauline Zheng, Sumi S Nair, Stacy C Bailey, Marina Serper, Peter P Reese, Daniela P Ladner, Michael S Wolf

**Affiliations:** 1 Center for Applied Health Research on Aging Feinberg School of Medicine Northwestern University Chicago, IL United States; 2 Mayo Clinic Arizona Transplant Center Mayo Clinic Pheonix, AZ United States; 3 Division of Gastroenterology and Hepatology Perelman School of Medicine University of Pennsylvania Philadelphia, PA United States; 4 Renal-Electrolyte and Hypertension Division Perelman School of Medicine University of Pennsylvania Philadelphia, PA United States; 5 Northwestern University Transplant Outcomes Research Collaborative Feinberg School of Medicine Northwestern University Chicago, IL United States; 6 Division of Transplant Department of Surgery Northwestern Medicine Chicago, IL United States

**Keywords:** kidney transplantation, medication adherence, fidelity, digital health, patient portal

## Abstract

**Background:**

Inadequate adherence to prescribed immunosuppressive medication regimens among kidney transplant recipients is common, yet interventions are needed to support patients in sustaining adequate adherence to prescribed regimens and achieving optimal transplant outcomes.

**Objective:**

We examined the preliminary fidelity of a transplant center-based, multifaceted adherence monitoring strategy known as TAKE IT.

**Methods:**

The TAKE IT strategy includes: (1) routine, online, monthly patient self-report adherence assessments; (2) care alerts directed to nurses; (3) quarterly reports monitoring tacrolimus values and adherence trends; (4) support tools tailored to specific adherence concerns. A 2-arm, patient-randomized trial is underway at two large transplant centers (N=449). To evaluate the initial fidelity of TAKE IT, we investigated patient uptake of monthly adherence assessments during the course of a 3-month period, whether any disparities emerged, and the nature of any reported adherence concerns.

**Results:**

Among 202 patients randomized and exposed to TAKE IT for 3-months or more, 81% (164/202) completed an adherence assessment, 73% (148/202) completed at least two, and 57% (116/202) completed all monthly assessments. Overall, 50% (82/164) of kidney transplant recipients reported at least one adherence concern over the 3-month assessment period. The most common barriers were classified as regimen-related (eg, regimen complexity), cognitive (eg, forgetfulness), and medical (eg, side effects). Higher-income participants were more likely to complete all surveys compared to lower-income participants (*P*=.01).

**Conclusions:**

TAKE IT demonstrated 81% (164/202) completion of an adherence assessment, 73% (148/202) completion of at least two, and 57% (116/202) completion of all monthly assessments during this brief, initial observation period. Among those that did respond to the online assessments, the majority demonstrated sustained engagement. Additional monitoring modalities could also be offered to meet patient preferences to ensure all patients’ medication use can be properly monitored.

**Trial Registration:**

ClinicalTrials.gov NCT03104868; https://clinicaltrials.gov/ct2/show/NCT03104868

## Introduction

Kidney transplant (KT) recipients require chronic immunosuppression to counteract graft rejection. However, inadequate medication adherence is a major cause of organ (graft) failure, with rates up to 48% post-transplant and even higher among at-risk patients (eg, racial/ethnic minorities, older adults, and those with poor health literacy) [1,2]. Poor adherence to the immunosuppression regimen is particularly high in KT patients (approximately 35%) compared to other organ transplant recipients [3-5]. Though current literature has indicated interventions, such as support tools (eg, reminder systems), monitoring strategies, and continuing education, to improve adherence, studies have largely been underpowered to provide conclusive evidence of their effectiveness [6-13]. Furthermore, medication adherence may be variably evaluated as part of post-transplant care. While immune suppression levels are measured as trough levels and provide a possible proxy indication of adherence specific to immunosuppression regimens, patient-reported assessments of any adherence concerns to the entire regimen one may be taking are not routinely embedded in clinical practice. Previous research has also found that medical staff has difficulty identifying patient adherence problems and factors driving suboptimal adherence [14]. Due to the complexity of factors that influence medication use, which may evolve over time, there has been increasing interest in finding ways to routinely monitor patients’ medication experiences and any potential barriers.

Digital health solutions, in particular, have been investigated as potential methods to surveil and address medication adherence [11,15,16]. There has been promising preliminary results and positive attitudes towards mobile health or web-based interventions to support regimen use, although further research is still needed to best understand how to integrate technologies into clinical workflows in an acceptable manner, both for patients and their care teams [17,18].

In 2017, the Transplant Regimen Adherence for Kidney recipients by Engaging Information Technologies, also known as TAKE IT, was launched to address inadequate regimen adherence among KT patients. The TAKE IT trial aims to address patient engagement and self-management with all prescribed medication regimens, not limited to immunosuppressants, by leveraging a web-based patient portal and a transplant centers’ electronic health record (EHR) to educate patients on their medication regimen, assist patients in organizing their daily prescription schedule efficiently, routinely monitor medication use, and provide care alerts to transplant center clinical staff when medication concerns are detected to mobilize the care and provide a response tailored to the specific concerns. It should also be mentioned that a prior trial, conducted in Canada and also known as TAKE IT, tested the effectiveness of an intervention to promote medication adherence among adolescent kidney recipients [11].

At the time of writing, TAKE IT is underway as a pragmatic, randomized clinical trial to test its effectiveness, compared to usual care, among diverse KT recipients. Primary outcomes related to medication-taking behaviors and regimen adherence, collected at 6 and 13 months, have now been completed. While evaluations of intervention effectiveness have not yet been performed, in the meantime, we sought, as planned, to examine the initial fidelity of the intervention’s ability to engage KT recipients beyond the point of care through the use of monthly invitations to complete brief portal assessments that allowed them to report on their regimen adherence and any specific concerns.

As some patients may lack the technological proficiency to interact with the online portal or may not be comfortable sharing details on their medication-taking behaviors with their healthcare providers, this investigation would inform which patients may not be adequately monitored in this manner. Thus, alternative methods may need to be offered by transplant centers.

## Methods

### Patients

The TAKE IT trial is a 2-arm, patient-randomized controlled trial conducted at two large tertiary care hospitals (Northwestern University and Mayo Clinic), which have a high volume of KT recipients annually. Patients within 5 weeks to 2 years post-transplant were recruited and were followed for 2 years. In-person baseline interviews were conducted, with telephone interviews given 6 weeks and 6 months post-baseline and in-person interviews at 12 months and 18 months post-baseline.

### Study Population

KT patients were eligible if they were 21 years of age or older, within 5 weeks to 24 months of KT, English-speaking, primarily responsible for administering their own medication, owned a cell phone and were comfortable receiving text messages, and had access and proficiency using the internet at home. This time interval of KT eligibility (ie, 5 weeks to 24 months post-transplant) was determined by prior studies indicating that adherence issues persist at different time points post-transplant, including early nonadherence, whether intentional or unintentional, due to regimen complexity, side effects, and health literacy [19-21]. Patients were excluded if they had severe, uncorrectable vision, hearing impairments, or cognitive impairments. For the purpose of this paper, fidelity data from patients in the intervention arm only were investigated.

### Intervention

Eligible participants were randomized to either intervention (TAKE IT) or usual care. The TAKE IT intervention is multifaceted and leverages a transplant center’s existing resources, including a routine monthly adherence assessment that requests patients to periodically self-report on their medication use. While self-reports of medication use and adherence may be biased due to socially desirable responses, more objective measures would be cost-prohibitive for routine use. Further, timely access to pharmacy fill data has also been problematic for most health systems and can also be inaccurate. Although this analysis will focus on evaluating the fidelity of TAKE IT’s monthly adherence assessments that request patients to periodically report on their medication use, the intervention strategy also includes additional components: (1) automated care alert notifications via the EHR identifying adherence-related problems to the transplant center nurse coordinator, (2) quarterly adherence reports that automatically calculate patient whole blood tacrolimus levels for transplant nurse coordinators, and (3) standardized protocols for mobilization of appropriate clinicians and staff for appropriate, tailored clinical support of existing transplant center tools to directly target adherence-related concerns identified by the results of routine TAKE IT adherence assessment. Specifically, if an adherence concern were to be identified, the appropriate care team member (eg, nurse, pharmacist, social worker, psychologist, etc.) would be expected to respond. Usual care refers to the normal standard clinical practices in place at either site, immediately post-transplant.

**Figure 1:**
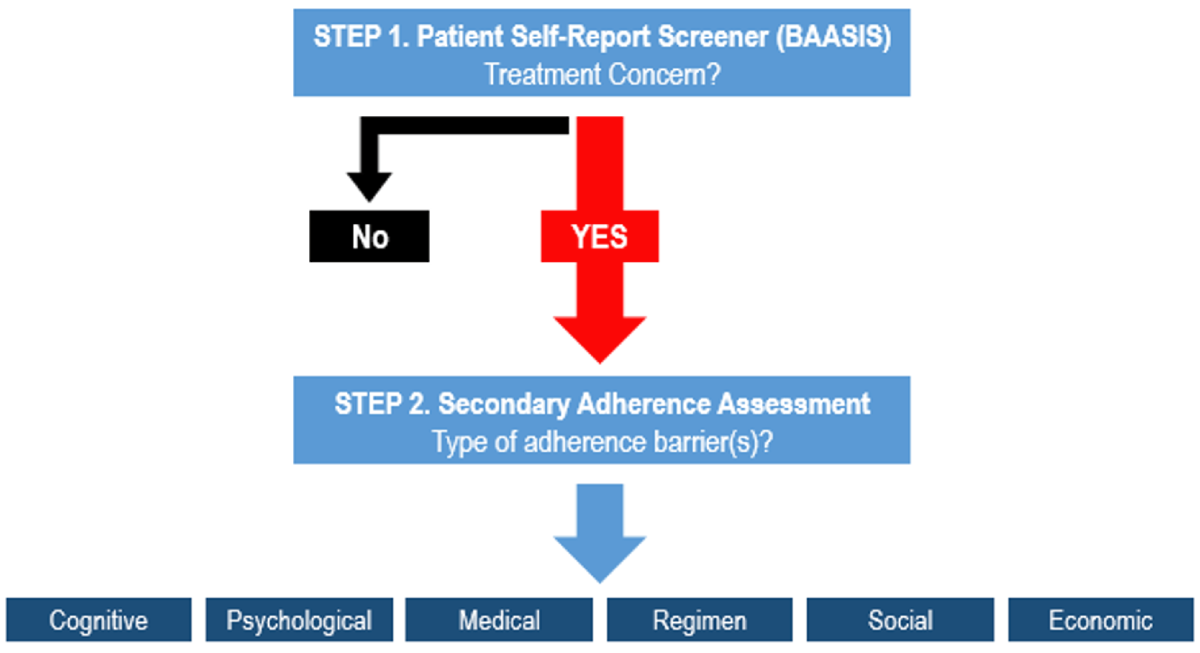
Flowchart of TAKE IT adherence assessment.

Patients in the TAKE IT intervention report their medication use on a monthly basis via the patient portal. This enables a continuous link between patients and the transplant center beyond routine in-person visits. The monthly, patient-reported adherence assessment includes a two-step approach to first determine a KT recipients’ regimen adherence status (adequate vs. inadequate). For this first step, we utilized the validated Basel Assessment of Adherence with Immunosuppressive medication Scales (BAASIS) instrument and a generated coefficient of variance from their last consecutive set of three tacrolimus levels. If patients self-report any adherence concerns via the BAASIS, they then complete a brief survey of items that capture the more specific nature of the adherence barriers. During this second step, a number of brief assessments seek to “phenotype” the presenting barrier(s) to further inform a transplant center’s response and deployment of resources. Thus, the monthly survey not only identifies patients at risk of inadequate adherence but also categorizes the nature of adherence concerns into the following: cognitive, psychological, medical, regimen, social, and economic (Figure 1).

Cognitive barriers include forgetfulness, memory issues, and difficulties concentrating and staying organized. Psychological barriers include low mood (eg, feeling down, depressed, hopeless) and little interest or pleasure in doing things. Medical barriers include side effects and self-reported overall health. Regimen barriers include confidence in taking medication as instructed, the complexity of regimen, missing medications, and taking medications earlier or later than instructed. Social barriers include issues around lack of support from family or healthcare providers. And finally, economic issues included issues around the cost of medications or refills. While not exhaustive, monthly survey items were taken from previously validated measures across these six categories, including self-reported assessments of cognitive complaints (eg, Brief Test of Adult Cognition by Telephone), depression (eg, Patient-Reported Outcomes Measurement Information System Depression subscale), regimen complexity (eg, Medication Regimen Complexity Index), unmet social needs (eg, Tangible Support Survey), and medication trade-offs, a survey assessing difficulties with medication affordability [22-28]. Given the potential risks of inadequate adherence, the study team defined the presence of a “concern” if participants endorsed any single item within the adherence assessment category.

### Procedure

KT patients were initially screened at both sites. Eligible patients were identified through EHRs. Participants who were eligible and interested in the study were scheduled for a baseline interview around their upcoming clinic visit, where applicable, with written consent obtained at the beginning of baseline interviews. At each site, enrolled patients were randomized by a 1:1 scheme to intervention (TAKE IT) or usual care. Patients complete the baseline interview at the transplant clinic in person. Starting 1-week post-baseline, TAKE IT participants complete the monthly, online, two-step adherence assessment. Any adherence concerns flagged during the monthly assessment will be included in a lab report that is sent to a designated clinic contact at each site immediately after the assessment is submitted. The report contains the patient’s identifying information, the nature of the adherence concern reported, and a recommendation for follow-up. The study was conducted in accordance with the approved IRB protocol (STU00204465).

### Measurement

To evaluate the initial fidelity of the TAKE IT strategy, we investigated what proportion of participants in the intervention arm completed online adherence assessments and described the prevalence and nature of adherence concerns to date. We restricted the sample for this analysis to only those participants who had received at least 3 consecutive monthly portal surveys, as this would allow us to examine their willingness to repeatedly complete the surveys. Specifically, survey completion over a 3-month timeline was first examined by the number of portal surveys completed (0 to 3 surveys) and then coded as an ordinal variable with 3 levels: 0 surveys completed, 1 or 2 surveys completed, and all surveys completed. The outcome of adherence concerns was categorized as a binary variable (yes/no to any adherence concern) per each of the six categories. The average number of adherence concerns identified from all surveys and the number of participants who flagged for more than two adherence concerns during any survey were also examined.

To assess any demographic disparities between participants across survey completion rates, patient characteristics were evaluated through a sociodemographic/health questionnaire. Age, days since transplant, and patient activation (as measured by the Consumer Health Activation Index or CHAI) were assessed as continuous variables. Gender was assessed as a binary variable (male or female). Health literacy was measured through the Newest Vital Sign and coded as a binary variable (inadequate or adequate). Global health was coded as a categorical variable with four ordinal levels: excellent, very good, good, and fair/poor. Ethnicity (ie, Hispanic) was coded as a binary variable (yes or no). Race was assessed as a three-category variable (White/Caucasian, Black/African American, and other). Education was coded as a three-category variable (less than college, some college or technical school, and college graduate). Income was also coded as a three-category variable (<US $30,000, US $30,000-US $49,999, and >US $50,000).

### Analysis Plan

Statistical analysis was conducted using RStudio (version 3.6.1; R Core Team). Appropriate descriptive statistics (eg, percentage, frequency, and median) were performed on all patient variables. Bivariate analysis was conducted to determine if there were any statistically significant demographic disparities between survey completion groups within the intervention arm. For categorical variables, data were analyzed using chi-square tests or Fisher exact test when expected cell counts were less than 5. For continuous variables (ie, age, CHAI scaled, and days since transplant), normality was assessed using the Shapiro test. No continuous variable was normally distributed; thus, bivariate analysis was conducted via Kruskal-Wallis, a nonparametric test that compares medians.

## Results

### Overview

Of the 449 participants enrolled in the TAKE IT trial, 224 (49.9%) participants were randomized to the intervention arm and analyzed for this investigation. Sociodemographic and clinical characteristics of this subsample are presented in Table S1 in Multimedia Appendix 1. Overall, the median age of intervention participants was 53 years (range 21-76), 58.4% (129/221) were male, and 19.2% (43/224) were African American. The median time since transplantation for these KT recipients was less than a year (202 days, range 23-1,091).

The majority of recipients (148/224, 66.1%) completed the initial online adherence assessment; there were no significant differences between participants who completed or did not complete the initial assessment in age, gender, race, or time since transplantation. However, participants who did not complete the initial assessment had significantly lower education (*P*=.02) and household income (*P*=.006; Table S1 Multimedia Appendix 1). Among those who did complete it, 34.6% (56/162) had one or more adherence concerns. The most common barriers were classified as regimen-related (25/56, 44.6%), cognitive (15/56, 26.8%), medical (11/56, 19.6%), and psychological (9/56, 16.1%).

### Repeat Completion of Monthly Portal Assessments

**Figure 2:**
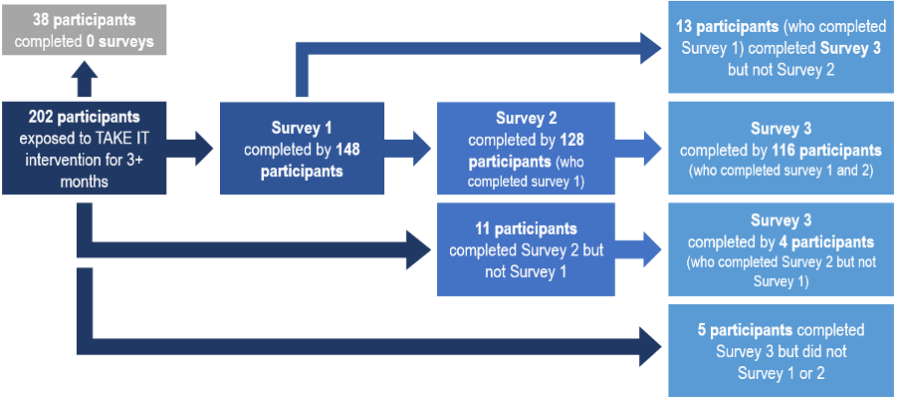
Flowchart of participant completion of TAKE IT adherence assessment.

We investigated repeat completion among 202 (90.2%) participants who had exposure to the intervention for three months or longer and thus had the chance to complete the initial online adherence assessment and 3-monthly follow-up surveys postbaseline. Intervention participants who had not been in the study for at least 3 months (22/224, 9.8%) were excluded from the analysis. Table S1 in Multimedia Appendix 1 provides sociodemographic characteristics of intervention patients exposed for three months or longer, stratified by survey completion. Overall, 81.2% (164/202) completed at least one assessment, and 73.3% (148/202) completed at least two assessments. Most (116/202, 57.4%) participants did complete all three surveys. There were no significant differences in age (*P*=.49), gender (*P*=.22), race (*P*=.50), education (*P*=.07), time since transplant (*P*=.94), health activation (*P*=.14), or health literacy (*P*=.30) between participants who completed no surveys, 1-2 surveys, or all surveys. Overall, participants who completed all surveys were more likely to have a higher income (*P*=.01) than participants who completed none or just one or two surveys. Figure 2 provides a flowchart illustrating the participants’ detailed completion of each of the monthly adherence assessments in time order.

Half of the intervention participants were at risk for inadequate adherence at some point over the three-month assessment period (Table 1). Among participants who completed 1 survey (16/202, 7.9%) versus 2 surveys (32/202, 15.8%) versus all 3 surveys (116/202, 57.4%), the proportion of those flagging for an adherence concern was 43.8% (7/16), 59.4% (19/32), and 48.3% (56/116), respectively. Among all of those who were identified as at risk for inadequate adherence, the average number of adherence concerns was 1.13, with a range of 1 to 5 adherence concerns. There were 26 (31.7%) participants who flagged for 2 or more adherence concerns. The most commonly reported barriers were cognitive (42/93, 45.2%), regimen-related (26/93, 28.0%), and medical (25/93, 26.9%) due to overall health or medication side effects).

The median time for participants who were sent and completed survey 1 (162/202, 80.2%) to open and return the assessment was 0.88 days (Table 2). A total of 51.9% (84/162) of participants completed the survey in less than 1 day, and among these recipients, the median time to return the assessment was 2.86 hours.

**Table 1:** Nature of adherence concerns (N=164).^a^

Participant characteristics		n (%)
**Any adherence concern**		
	No	82 (50)
	Yes	82 (50)
Flagged for 2+ concerns		26 (31.7)
Total adherence concerns		93 (56.7)
**Adherence concern type**		
	Cognitive	42 (45.2)
	Regimen	26 (28.0)
	Medical	25 (26.9)
	Psych	16 (17.2)
	Economic	5 (5.4)
	Social	4 (4.3)

^a^An average of 1.13 concerns.

**Table 2:** Time between sending survey 1 and completing survey 1 (N=162).

Survey 1 characteristics	Values
Time to completion (days), mean (SD)	2.52 (3.55)
Time to completion (days), median (range)	0.88 (1.22 mins-23.06 days)
Participants completing survey in > 1 day, n(%)	84 (51.9%)
Time to completion (hours), mean (SD)	4.78 (5.86)
Time to completion (days), median (range)	2.86 (1.22 mins-23.17 hrs)

## Discussion

### Principal Findings

In this diverse sample of KT recipients, there was albeit modest, while relatively high uptake to initially responding to the monthly adherence assessments using the patient portal, yet sustained engagement over the 3-month period among those who did respond with the monthly portal assessment. Of the 202 intervention participants who had the chance to complete the initial online adherence assessment and three monthly follow-up surveys postbaseline, most (n=164/202, 81%) completed at least one assessment, and 57% (116/202) completed all assessments. Further, the flowchart of participants’ completion of monthly adherence assessments (Figure 2) illustrated KT patients who demonstrated intermittent engagement (eg, did not complete survey 1 but completed surveys 2 and 3) versus sustained engagement to all monthly assessments. Though this may be due to the study’s limitation of a 3-month analysis, this may also point to challenges in survey complexity, patient indifference to completing monthly surveys, or more specific patient-level barriers in survey completion, such as time or forgetfulness. The results of monthly assessment engagement may also suggest that, perhaps, the frequency of assessments may benefit from shifting from monthly surveys to bimonthly or quarterly surveys. In technology-based intervention studies targeting medication adherence in transplant recipients, McGillicuddy et al [29] and Taber et al [30] managed to receive 91% and 98% retention, respectively; however, the McGillicuddy intervention engagement was passive, and both studies were small pilot samples of less than 68 participants in the intervention arm. Trends in sustained engagement to the TAKE IT monthly adherence assessments may be better clarified in future analyses as participants’ exposure to the intervention increases.

In addition to high retention, over half of participants (84/162, 51.9%) who were sent the first survey completed the survey in less than one day after the survey was sent to them, suggesting that the monthly surveys may not pose a huge burden on patients. Furthermore, medication adherence concerns assessed by TAKE IT’s monthly assessments align with results from a recent study examining barriers to immunosuppressant medication adherence in KT recipients [31]. This same study categorized regimen-specific barriers largely as delaying doses (70/156, 45%) and skipping doses (40/156, 25%), often from daily routine changes or other factors (eg, financial issues). Our study demonstrated a high prevalence of inadequate adherence (82/164, 50%), with adherence barriers targeted around cognitive, regimen-related, and medical issues (eg, side effects or overall health issues).

We also sought to investigate any differences in the receptiveness of TAKE IT intervention by patient factors. Only lower income was associated with statistically significantly lower uptake in TAKE IT assessments. While the reasons are unclear, it could be speculated that lower uptake of the monthly assessments might be an access issue. If a patient’s internet access is primarily through their mobile phone use, it will put participants without a laptop or desktop at a disadvantage. The significance of lower income on assessment uptake may also point to psychosocial trade-offs. For example, a low-income patient may experience more psychosocial stress (eg, working multiple jobs) and not have time to prioritize surveys.

Though low income was the only significant factor in survey completion, it should be noted that non-significant differences were observed in other factors, including health activation, health literacy, global health, ethnicity (ie, Hispanic), and education. Again, reasons are unclear if these point to an access or use issue, but it could be that patients who are not as activated (or motivated) or who have greater difficulty understanding health information (eg, prescription information) because they have lower health literacy or lower education may not be as responsive to monthly adherence surveys. Future studies including a larger transplant sample may be better powered to investigate the impact of health activation, health literacy, and education on study assessment completion.

As of now, there were no significant differences overall in participant characteristics related to known risk factors for medication adherence (eg, age, time since transplant, race). These results are promising and possibly suggest that the TAKE IT intervention might not create further disparities and may provide equitable solutions across patient groups; however, further research should examine whether this strategy can, in fact, work among diverse populations.

### Limitations

There were several other limitations of the TAKE IT fidelity analysis. First, analysis was limited to an intervention follow-up of 3 months. Currently, we are unable to assess if the TAKE IT strategy is impactful on changing adherence behaviors or outcomes in the long-term, as active follow-up is ongoing; however, future analyses may elucidate long-term outcomes. Second, TAKE IT assessments are optimized for desktop and laptop use rather than mobile phones. As mentioned above, this potentially poses an access barrier for participants who do not have easy or reliable access to a desktop or laptop. Third, study participants were recruited from two large tertiary care hospitals, and fourth, intervention excludes participants who do not speak English—both of which limit the generalizability of findings.

### Conclusions

Overall, the TAKE IT trial demonstrates 81% (164/202) completion of an adherence assessment, 73% (148/202) completion of at least two, and 57% (116/202) completion of all monthly assessments among diverse patient groups, notably across age, time since transplant, health literacy, and race—a potential benefit in monitoring adherence behaviors among KT recipients and engaging transplant center staff to address at-risk patients. Assessments of medication adherence and potential root causes of poor adherence, including psychological and social determinants, can be captured beyond the point of care via brief, routine online surveys via an EHR patient portal. Such routine assessments have the potential for earlier detection of adherence concerns between clinic visits without adding to already busy clinical workloads. Future work should focus on deploying adherence support tools tailored to specific adherence concerns and addressing possible disparities in access to this technology-enabled strategy. The goal would be to ensure that both responding to online assessments and deploying support tools can be integrated into existing clinic workflows without burdening clinical staff. Furthermore, ensuring monthly surveys are mobile phone friendly and assessing TAKE IT among non-English speaking populations to clarify why TAKE IT may not be as acceptable to Hispanic populations would be important next steps to improve uptake. Additional work should also include qualitative research for understanding why participants do not complete surveys and evaluating clinicians’ perspectives if a patient’s nonresponse should be a clinical response in itself.

## Appendix

Multimedia Appendix 1 Characteristics of participants exposed to intervention for 3 months, by survey completion.

## Abbreviations

BAASIS: Basel Assessment of Adherence with Immunosuppressive medication Scales
CHAI: Consumer Health Activation Index
EHR: electronic health record
KT: kidney transplant
TAKE IT: Transplant Regimen Adherence for Kidney recipients by Engaging Information Technologies
