# Supplementary material for: A Multifaceted Intervention to Improve Medication Adherence in Kidney Transplant Recipients: An Exploratory Analysis of the Fidelity of the TAKE IT Trial
Source: JMIR Form Res. 2022 May 5;6(5):e27277. doi: 10.2196/27277 (PMC9121227; doi:10.2196/27277)
Supplement: Multimedia Appendix 1 [file formative_v6i5e27277_app1.docx]

This is a Multimedia Appendix to a full manuscript published in the J Med Internet Res. For full copyright and citation information see http://dx.doi.org/10.2196/jmir.27277

*Table S1. Characteristics of participants exposed to intervention for 3 months, by survey completion*

| **3 Surveys Sent N=202** | **All Participants**  **N=202** | | **0 Surveys Completed**  **n=38 (18.8%)** | | **1 or 2 Surveys Complete**  **n=48 (23.8%)** | | **All Surveys Complete**  **n=116 (57.4%)** | |  |
| --- | --- | --- | --- | --- | --- | --- | --- | --- | --- |
| **Participant Characteristics*** | **n** | **%** | **n** | **%** | **n** | **%** | **n** | **%** | **p-value** |
| **Age (median, range)** | 53 (21, 76) |  | 50 (22, 76) |  | 52 (25, 75) |  | 54 (21, 75) |  | 0.49 |
| **Days Since Transplant (median, range)** | 246 (23, 1,091) |  | 223.5 (44, 962) |  | 205 (23, 1,091) |  | 258 (32, 1,037) |  | 0.94 |
| **CHAI Scaled (median, range)** | 84 (50, 100) |  | 82 (50, 100) |  | 82 (56, 100) |  | 86 (56, 100) |  | 0.14 |
| **Gender** |  |  |  |  |  |  |  |  | 0.22 |
| Male | 120 | 59.7% | 18 | 47.4% | 31 | 64.6% | 71 | 61.7% |  |
| Female | 81 | 40.3% | 20 | 52.6% | 17 | 35.4% | 44 | 38.3% |  |
| **NVS (health literacy)** |  |  |  |  |  |  |  |  | 0.30 |
| Inadequate | 69 | 34.2% | 17 | 44.7% | 16 | 33.3% | 36 | 31.0% |  |
| Adequate | 133 | 65.8% | 21 | 55.3% | 32 | 66.7% | 80 | 69.0% |  |
| **Global Health** |  |  |  |  |  |  |  |  | 0.14 |
| Excellent | 22 | 10.9% | 4 | 10.5% | 1 | 2.1% | 17 | 14.7% |  |
| Very Good | 74 | 36.6% | 13 | 34.2% | 19 | 39.6% | 42 | 36.2% |  |
| Good | 79 | 39.1% | 13 | 34.2% | 20 | 41.7% | 46 | 39.7% |  |
| Fair/Poor | 27 | 13.4% | 8 | 21.1% | 8 | 16.7% | 11 | 9.5% |  |
| **Hispanic** |  |  |  |  |  |  |  |  | 0.14 |
| No | 167 | 83.1% | 28 | 73.7% | 43 | 89.6% | 96 | 83.5% |  |
| Yes | 34 | 16.9% | 10 | 26.3% | 5 | 10.4% | 19 | 16.5% |  |
| **Race** |  |  |  |  |  |  |  |  | 0.50 |
| White/Caucasian | 132 | 65.3% | 24 | 63.2% | 29 | 60.4% | 79 | 68.1% |  |
| Black/African American | 40 | 19.8% | 6 | 15.8% | 13 | 27.1% | 21 | 18.1% |  |
| Other | 30 | 14.9% | 8 | 21.1% | 6 | 12.5% | 16 | 13.8% |  |
| **Education** |  |  |  |  |  |  |  |  | 0.07 |
| Less than college | 46 | 22.9% | 11 | 28.9% | 12 | 25.0% | 23 | 20.0% |  |
| Some college or technical school | 69 | 34.3% | 17 | 44.7% | 19 | 39.6% | 33 | 28.7% |  |
| College graduate | 86 | 42.8% | 10 | 26.3% | 17 | 35.4% | 59 | 51.3% |  |
| **Income** |  |  |  |  |  |  |  |  | 0.01** |
| < $30,000 | 54 | 27.7% | 17 | 44.7% | 13 | 27.1% | 24 | 22.0% |  |
| $30,000 to $49,999 | 28 | 14.4% | 8 | 21.1% | 7 | 14.6% | 13 | 11.9% |  |
| > $50,000 | 113 | 57.9% | 13 | 34.2% | 28 | 58.3% | 72 | 66.1% |  |
| * values may not add up to total sample size, due to participants with missing demographic information | | | | | | | | | |
| ** indicates statistically significant differences between groups (p-value < 0.05) | | | | | | | | | |
